# Correction: Ishaque et al. Iriflophenone-3-C-β-d Glucopyranoside from *Dryopteris ramosa* (Hope) C. Chr. with Promising Future as Natural Antibiotic for Gastrointestinal Tract Infections. *Antibiotics* 2021, *10*, 1128

**DOI:** 10.3390/antibiotics11030301

**Published:** 2022-02-24

**Authors:** Muhammad Ishaque, Yamin Bibi, Samha Al Ayoubi, Saadia Masood, Sobia Nisa, Abdul Qayyum

**Affiliations:** 1 Department of Botany, PMAS-Arid Agriculture University Rawalpindi, Rawalpindi 46300, Pakistan; mishaque270@gmail.com (M.I.); dryaminbibi@uaar.edu.pk (Y.B.); 2 College of Humanities and Sciences, Prince Sultan University, Rafha Street, Riyadh 11586, Saudi Arabia; sayoubi@psu.edu.sa; 3 Department of Statistics & Mathematics, PMAS-Arid Agriculture University Rawalpindi, Rawalpindi 46300, Pakistan; 4 Department of Microbiology, The University of Haripur, Haripur 22620, Pakistan; sobia@uoh.edu.pk; 5 Department of Agronomy, The University of Haripur, Haripur 22620, Pakistan

At the request of Dr. Markus Bacher, Dr. Johann Schinnerl, and Dr. Karin Valant-Vetschera, they have been removed as authors of the paper [1] because their agreement was not obtained for its publication in the present form. The remaining authors indicate that this change does not affect the scientific conclusions.

Author Contributions should be:

**Author Contributions:** M.I. and Y.B. conceptualized. M.I. performed experimentation; Y.B. supervised the study, S.N. analyze the data. S.M. analyze the data through statistical tools. S.A.A. and A.Q. involved in preparation of original draft. Y.B. reviewed the draft. All authors have read and agreed to the published version of the manuscript.
